# Evolution and Innovations in Bone Marrow Cellular Therapy for Musculoskeletal Disorders: Tracing the Historical Trajectory and Contemporary Advances

**DOI:** 10.3390/bioengineering11100979

**Published:** 2024-09-28

**Authors:** José Fábio Lana, Gabriela Caponero de Brito, André Kruel, Benjamim Brito, Gabriel Silva Santos, Carolina Caliari, Francesca Salamanna, Maria Sartori, Giovanni Barbanti Brodano, Fábio Ramos Costa, Madhan Jeyaraman, Ignácio Dallo, Pedro Bernaldez, Joseph Purita, Marco Antonio Percope de Andrade, Peter Albert Everts

**Affiliations:** 1 Department of Orthopaedics, Brazilian Institute of Regenerative Medicine (BIRM), Indaiatuba 13334-170, SP, Brazil; josefabiolana@gmail.com (J.F.L.); gabriela_caponero@hotmail.com (G.C.d.B.); kruel.andre@gmail.com (A.K.); contato@clinica4move.com.br (B.B.); 2 Regenerative Medicine, Orthoregen International Course, Indaiatuba 13334-170, SP, Brazil; doctorignaciodallo@gmail.com (I.D.); jpurita@aol.com (J.P.); everts@me.com (P.A.E.); 3 Medical School, Max Planck University Center (UniMAX), Indaiatuba 13343-060, SP, Brazil; 4 Clinical Research, Anna Vitória Lana Institute (IAVL), Indaiatuba 13334-170, SP, Brazil; 5 Medical School, Jaguariúna University Center (UniFAJ), Jaguariúna 13820-000, SP, Brazil; 6 Cell Therapy, In Situ Terapia Celular, Ribeirão Preto 14056-680, SP, Brazil; caliari.carolina@gmail.com; 7 Surgical Sciences and Technologies, IRCCS Instituto Ortopedizo Rizzoli, 40136 Bologna, Italy; francesca.salamanna@ior.it (F.S.); maria.sartori@ior.it (M.S.); 8 Spine Surgery Unit, IRCCS Instituto Ortopedizo Rizzoli, 40136 Bologna, Italy; giovanni@barbantibrodano.com; 9 Department of Orthopaedics, FC Sports Traumatology, Salvador 40296-210, BA, Brazil; fabiocosta123@uol.com.br; 10 Department of Orthopaedics, ACS Medical College and Hospital, Dr. MGR Educational and Research Institute, Chennai 600077, Tamil Nadu, India; madhanjeyaraman@gmail.com; 11 Orthopaedic Research Group, Coimbatore 641045, Tamil Nadu, India; 12 Clinical Research Scientist, Virginia Tech India, Chennai 600095, Tamil Nadu, India; 13 Orthopedics, SportMe Medical Center, 41013 Seville, Spain; pedrobernaldez@gmail.com; 14 Department of the Locomotor Apparatus, Federal University of Minas Gerais, Belo Horizonte 31270-901, MG, Brazil; mapa.bhz@terra.com.br; 15 Gulf Coast Biologics, Fort Myers, FL 33916, USA

**Keywords:** bone marrow, orthopedics, mesenchymal stem cells, regenerative medicine

## Abstract

Bone marrow cellular therapy has undergone a remarkable evolution, significantly impacting the treatment of musculoskeletal disorders. This review traces the historical trajectory from early mythological references to contemporary scientific advancements. The groundbreaking work of Friedenstein in 1968, identifying fibroblast colony-forming cells in bone marrow, laid the foundation for future studies. Caplan’s subsequent identification of mesenchymal stem cells (MSCs) in 1991 highlighted their differentiation potential and immunomodulatory properties, establishing them as key players in regenerative medicine. Contemporary research has focused on refining techniques for isolating and applying bone marrow-derived MSCs. These cells have shown promise in treating conditions like osteonecrosis, osteoarthritis, and tendon injuries thanks to their ability to promote tissue repair, modulate immune responses, and enhance angiogenesis. Clinical studies have demonstrated significant improvements in pain relief, functional recovery, and tissue regeneration. Innovations such as the ACH classification system and advancements in bone marrow aspiration methods have standardized practices, improving the consistency and efficacy of these therapies. Recent clinical trials have validated the therapeutic potential of bone marrow-derived products, highlighting their advantages in both surgical and non-surgical applications. Studies have shown that MSCs can reduce inflammation, support bone healing, and enhance cartilage repair. However, challenges remain, including the need for rigorous characterization of cell populations and standardized reporting in clinical trials. Addressing these issues is crucial for advancing the field and ensuring the reliable application of these therapies. Looking ahead, future research should focus on integrating bone marrow-derived products with other regenerative techniques and exploring non-surgical interventions. The continued innovation and refinement of these therapies hold promise for revolutionizing the treatment of musculoskeletal disorders, offering improved patient outcomes, and advancing the boundaries of medical science.

## 1. Introduction

The use of bone marrow-derived products emerged in the search for tissue repair aimed at preserving and enhancing the functionality of tissues and organs. Regenerative medicine has shown promising and significant progress over the last decades in the treatment of musculoskeletal disorders, with bone marrow-derived products standing out as an effective and non-invasive tool for managing these comorbidities [1,2].

The investigation of bone marrow regenerative potential can be traced back to the groundbreaking work of Friedenstein et al. in 1968 [3], who identified the bone marrow-derived cells’ ability to form fibroblast colonies and differentiate into various tissue lineages. Their findings laid the foundation for subsequent research into cell-based therapies [4]. Since then, the field has expanded significantly, driven by a deeper understanding of bone marrow-derived cell properties and the development of advanced techniques for product isolation and application.

MSCs, first described by Caplan in 1991, can be obtained through the bone marrow, serving as a reservoir for these cells [2]. Apart from hematopoiesis, they play a vital role in various physiological processes. Mesenchymal cells exhibit considerable potential for tissue regeneration and repair, particularly in bone and cartilaginous tissues. This unique characteristic underscores their potential use in regenerative therapy [4]. Furthermore, bone marrow aspirate (BMA) and bone marrow aspirate concentrate (BMAC) have been applied in various orthopedic conditions. While BMA is employed without manipulation, BMAC undergoes centrifugation. Both have shown promising results in the treatment of osteoarthritis, rotator cuff injuries, osteonecrosis, and pseudarthrosis, with reported improvements in pain, functional repair in non-surgical procedures, and increased healing processes in surgical applications [5,6,7,8,9,10]. This article aims to trace and delineate the historical evolution of bone marrow-derived products used in musculoskeletal disorders, tracing their origins to the latest advancements.

**Figure 1:**
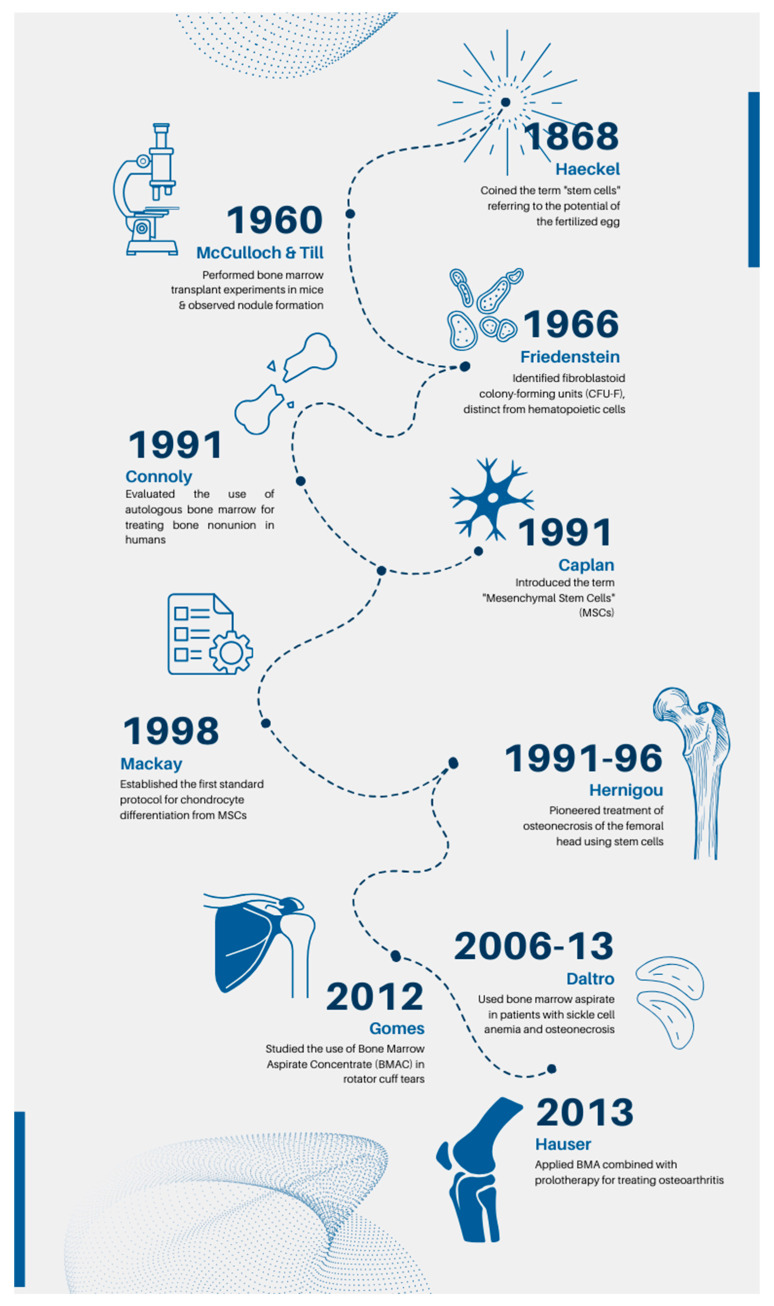
Main historical milestones and clinical advances in bone marrow cellular therapies.

The present overview outlines the trajectory of bone marrow-derived product utilization in musculoskeletal disorders, aiming to provide a comprehensive perspective on our historical journey (Figure 1) and potential future directions in applying regenerative strategies within the realm of orthopedic pathologies.

This review will be structured to provide a clear trajectory of bone marrow-derived products from their historical origins to contemporary advancements and future prospects. The manuscript is organized into sections that address historical developments, current advancements, and potential future directions in the field.

### 1.1. Historical Development, First Concepts, and the Initial Applications of Bone Marrow Mesenchymal Stem Cells Therapies

The origins of regenerative medicine can be traced back to ancient Greece, where a myth involving Prometheus serves as an early reference to the concept of regeneration. According to the myth, Prometheus was punished by Zeus for giving fire to mortals. Bound to a rock, Prometheus endured the daily torment of having his liver eaten by an eagle, only to have it regenerate overnight, symbolizing the idea of perpetual renewal. The term “stem cells” was first described in 1868 by Ernst Haeckel, who referred to the fertilized egg’s capability to develop into various cell types within a living organism [11].

### 1.2. Scientific Milestones

Interested in this potential of stem cells, scientists in the late 19th century began exploring the possibility of transplantation as a means of replacing damaged tissues. In the early 1960s, McCulloch and Till performed bone marrow transplant experiments in mice and observed nodule formation. Subsequently, along with Becker, they published an article confirming bone marrow cell capacity for self-renewal, a pivotal discovery for understanding stem cell functionality. They also demonstrated that bone marrow transplantation could reconstitute the hematopoietic system in mice, highlighting the potential of bone marrow cells to give rise to all types of blood cells, which they termed hematopoietic stem cells [12].

Concurrently, E. Donnall Thomas pioneered the transplantation of hematopoietic cells from bone marrow as a viable treatment for leukemia. Through significant advancements and extensive transplantation procedures, he described how these cells could regenerate a patient’s hematologic system [13,14].

In 1966, Friedenstein et al. observed the presence of a cell distinct from hematopoietic cells, possessing specific characteristics and the ability to form colonies. This discovery led to the coining of the term “fibroblastoid colony-forming units” (CFU-F) [3]. The first use of the term “Mesenchymal Stem Cell” (MSC) was introduced by Caplan in 1991, highlighting their immunomodulatory functions and their potential to differentiate into various cell types. MSCs were also noted for their paracrine and signaling actions, prompting an update to the term “medicinal signaling cells”, according to Caplan [4,15].

**Figure 2:**
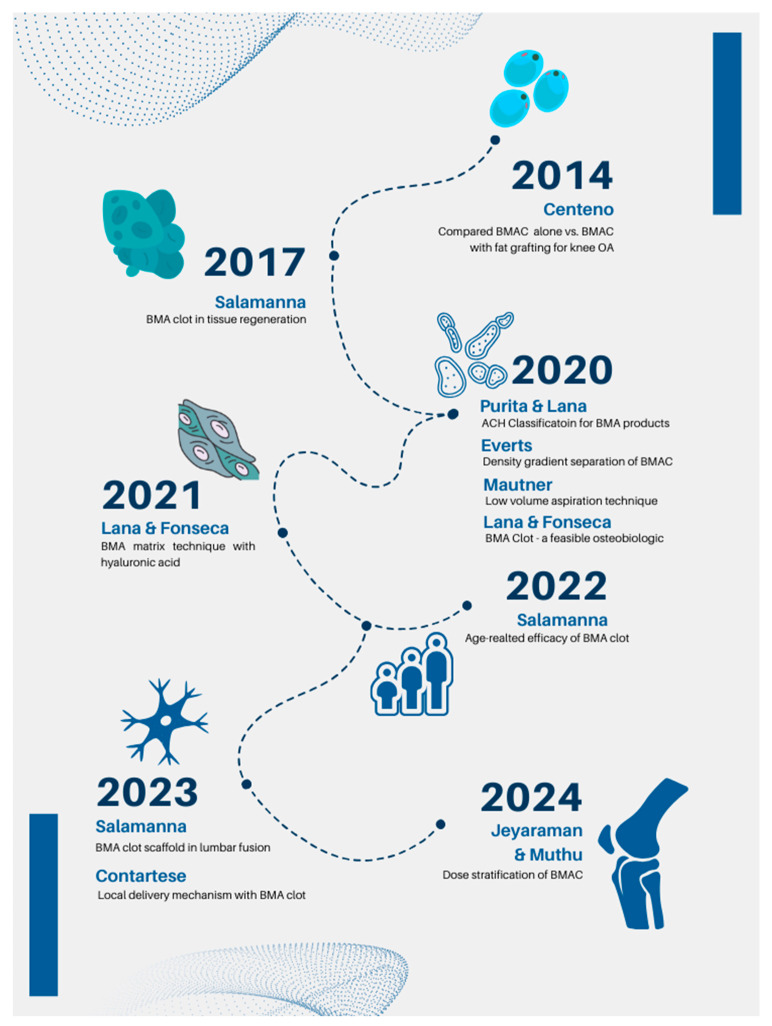
Recent clinical advances in the bone marrow cellular therapies.

Lindholm and Urist were the pioneers in describing the use of unprocessed bone marrow aspirate (BMA) alongside allograft bone matrix to augment bone healing [16]. Their groundbreaking work was subsequently built upon by Connolly and Shindell, who reported favorable outcomes with injections of unprocessed BMA alone for percutaneous treatment of tibial nonunion. Since these seminal studies, both BMA and materials derived from concentrated bone marrow have been harnessed for treating a wide spectrum of musculoskeletal conditions. Hernigou pioneered the treatment of osteonecrosis of the femoral head using bone marrow MSCs from 1990 to 1996. He conducted a study involving 189 patients with osteonecrosis of the femoral head to evaluate the effectiveness of concentrated bone marrow aspirate in treating this condition. The product, obtained from the anterior iliac crest, was applied for the repair and reshaping of the femoral head, with the aim of delaying or avoiding the need for hip arthroplasty. Patient monitoring for 5 to 11 years underscored the potential of progenitor cells or growth factors in optimizing bone repair and maintaining femoral head integrity. The study also indicated that the treatment outcome could be influenced by the quantity of transplanted progenitor cells, with hips receiving a higher number of cells showing better outcomes. These findings provided significant evidence of the potential of stem cell-based therapy for the treatment of bone nonunion [6]. The historical milestones are given in Figure 2.

## 2. Biological Characteristics of Bone Marrow Mesenchymal Stem Cells

Bone marrow MSCs primarily reside within marrow cavities, though they are occasionally found around blood vessels or other tissues. In 2006, the International Society of Cellular Therapy (ISCT) established four minimum criteria to define mesenchymal stem/stromal cells (MSCs), comprising fibroblast-like morphology, plastic adherence, multilineage and multipotential capacity for differentiation into osteoblasts, adipocytes, and chondrocytes. These cells should also express the cell surface proteins CD73, CD90, and CD105, while lacking expression of the lineage-specific markers CD45, CD34, CD14, CD19, CD11b, and HLA-DR. More recently, in 2019, Viswanathan et al. published a seminal paper aimed at elucidating the diverse nomenclature associated with mesenchymal stromal cells (MSCs) versus mesenchymal stem cells (also abbreviated as MSC). The research group critically analyzed the previously outlined criteria for defining MSCs, particularly noting that these criteria primarily apply to in vitro expanded MSCs. They highlighted the complexity surrounding the expression of surface markers CD34 and HLA-DR molecules in vivo, revealing that MSCs can exhibit CD34 positivity under certain conditions, such as when exposed to Insulin-like Growth Factor 1 in culture medium. Additionally, they demonstrated that HLA-DR surface markers can be influenced by interactions with interferon gamma. Moreover, Viswanathan et al. underscored the imperative of precisely identifying the tissue of origin for MSC isolation due to observed differences in characteristics. They advocated for a more functional definition of MSCs versus mesenchymal stem cells, given the absence of definitive surface markers to distinguish between these cell populations. They elucidated that the term “mesenchymal stromal cells” encompasses a heterogeneous cell population comprising fibroblasts, myofibroblasts, and stem cells while excluding hematopoietic and endothelial cells. Thus, they recommended further characterization of MSC populations based on their functional profile in addition to phenotyping, alongside comparisons with appropriate references, such as MSCs in a resting state. In conclusion, Viswanathan et al. stressed the importance of maintaining a clear distinction between stem cells and stromal cells based on their functional abilities and characteristics. They proposed that the term “mesenchymal stem cells” should only be utilized when tri-lineage differentiation potential is demonstrated both in vivo and in vitro.

MSCs derived from bone marrow are considered the most widely used and exhibit all the typical characteristics of MSCs; consequently, their derivatives exhibit considerable immunoregulatory potential. This potential has been reiterated in several studies [17,18]. MSCs may decrease the activation and differentiation of dendritic cells, polarize the differentiation of M0 macrophages to the M2 subtype [18,19], reduce the proliferation of T and B lymphocytes, and promote polarization toward regulatory T lymphocytes (Treg) and regulatory B lymphocytes [17,18]. These characteristics are also advantageous in the context of musculoskeletal diseases, as interactions between bone and immune cells are crucial for metabolism and bone formation during tissue repair. Initial inflammation in musculoskeletal injuries involves the infiltration of neutrophils, macrophages, and lymphocytes as well as the release of inflammatory cytokines [20]. Furthermore, in vitro studies demonstrate that when induced to differentiate into bone, MSCs derived from bone marrow maintain their property of inhibiting the proliferation of lymphocytes; however, differentiation into chondrocytes alters this capacity [20].

Angiogenesis and neovascularization represent additional critical steps in the restoration of damaged tissues, and numerous studies highlight the significant potential of orthobiologics in stimulating this process [21]. In this context, it has been described that bone marrow endogenously produces vascular endothelial growth factor (VEGF) and other hematopoietic-derived cytokines, such as erythropoietin, stem cell factor (SCF), and granulocyte colony stimulator (G-CSF). Additionally, endothelial cells and pericytes act synergistically not only in the maintenance but also in the formation of new blood vessels [22].

Rodriguez-Menocal and colleagues [17] compared the effect of bone marrow aspirate, bone marrow mononuclear cells, and bone marrow mesenchymal cells in inducing angiogenesis in vitro. Superior angiogenesis was observed in the bone marrow aspirate group compared to the mononuclear or mesenchymal cells. The authors concluded that the mixture of bone marrow cells presents a powerful paracrine component capable of inducing angiogenesis more efficiently than isolated cells [23].

Over the past decade, advancements have also shown that bone marrow MSCs produce robust pain relief in a variety of conditions, ranging from neuropathic pain in mice to osteoarthritis in patients [24,25]. The immunosuppressive and immunomodulatory properties of stem cells have been shown to contribute to this pain relief [26,27]. Additionally, the secretome of stem cells is also sufficient to produce these effects, suggesting that the paracrine release of cytokines and other factors is necessary. Specifically, MSCs secrete anti-inflammatory cytokines, such as interleukin-10 (IL-10), transforming growth factor-β (TGF-β), and other immunomodulatory molecules and chemokines [13,28] and produce increased amounts of PGE2, which regulates cyclooxygenase 2/prostaglandin E2 signaling [29]. However, further investigations are warranted to study the MSCs’ capabilities to suppress inflammation and optimize nociceptive effects. It is important to consider that many factors may influence pain outcomes, such as the type of MSCs used, the route of administration, and the specific immunomodulatory properties of applied MSCs [30]. The main advantage of using bone marrow cellular therapy is its ability to promote tissue regeneration and repair through the unique properties of mesenchymal stem cells (MSCs), offering effective, non-invasive, and minimally invasive treatment options for musculoskeletal disorders, thereby reducing pain, enhancing healing, and potentially avoiding surgical interventions.

## 3. Contemporary Era: Main Clinical Advances on Bone Marrow Mesenchymal Stem Cells Therapies in Humans

Following Hernigou’s pioneering work, other researchers published studies (Table 1) on the use of bone marrow aspirate for treating femoral osteonecrosis. From 2006 to 2013, Gildasio Daltro conducted a study involving 89 patients diagnosed with sickle cell anemia and osteonecrosis of the femoral head. These patients underwent clinical follow-up for 60 months post-procedure, showing significant improvement in pain symptoms, joint function, and disease stabilization. This study highlighted an important therapeutic tool for managing sickle cell disease and its common complication, femoral head osteonecrosis, especially prevalent in Brazil’s Bahia region [31]. At the same time, Ellera Gomes et al., in 2012, studied 14 patients with complete rotator cuff tears, treated conventionally through transosseous suturing with the addition of adjuvant bone marrow mononuclear cells (BMNC) at the site. Over 12 months of monitoring, patients showed functional improvements and, via magnetic resonance imaging, maintenance of tendon integrity. This study suggested that BMNC may offer additional benefits in repairing rotator cuff injuries, contributing to improved clinical and functional outcomes [10]. Subsequently, Tabataee et al., in 2015, conducted a comparative study demonstrating the effectiveness of combining bone marrow aspirate with decompression for treating initial necrosis [32].

### 3.1. Recent Clinical Trials and Studies

Between 2012 and 2015, Butala evaluated 10 cases of pseudarthrosis treated with unprocessed bone marrow, concluding that bone marrow aspirate offers advantages over other biological products, such as platelet-rich derivates, due to its simplicity, effectiveness, affordability, and ease of execution [43]. Anticipating improved outcomes in the treatment of joint diseases, Hauser et al. used BMA combined with prolotherapy (dextrose) to treat osteoarthritis of the hip, knee, and toe. All treated patients reported improvements, with a subset experiencing complete pain relief and restoration of function [44].

In 2015, Centeno et al. evaluated the efficacy of BMAC in treating glenohumeral osteoarthritis, with or without associated rotator cuff injury. Besides demonstrating BMAC’s safety, the outcomes in the 115 studied shoulders showed improvements in the Disability of Arm, Shoulder, and Hand (DASH) scores, the Visual Analog Scale (VAS) for pain, and subjective improvement as perceived by the patients [45]. Various augmentation methods have been used alongside BMAC, including adipose tissue grafts, platelet products, hyaluronic acid, and collagen matrices. All these studies aim to find an ideal scaffold for mesenchymal cells derived from BMAC. In 2014, Centeno conducted a comparative study for treating knee osteoarthritis using BMAC alone versus BMAC with fat grafting. Both approaches showed significant symptom improvement, but the addition of a fat graft did not yield more effective results than BMAC alone [7,38,46].

Salamanna, in 2017, conducted a pivotal systematic review on the use of a BMA clot, a spontaneously coagulated product derived from bone marrow and employed immediately without manipulation. The study highlighted the significance of the BMA clot as a scaffold for osteogenic cells, enhanced angiogenesis resulting from fibrinolytic activity within the clot, and its promising combination and synergy with growth factors and osteoinducers to augment tissue regeneration [33]. In 2017, Shapiro evaluated 25 patients with knee osteoarthritis, comparing the application of BMAC mixed with platelet-poor plasma (PPP) to saline solution. Despite not demonstrating significant results in pain relief or magnetic resonance imaging, BMAC was proven to be a viable, reliable, and extremely safe product without adverse effects [8].

Gianakos et al., in 2017, conducted a review to understand the use of BMAC in musculoskeletal diseases. They identified 36 studies involving BMAC, which covered various conditions including chondral lesions (7 studies), osteochondral lesions (10 studies), osteoarthritis (5 studies), nonunion or fracture (9 studies), and tendon injuries (5 studies). One finding of the study was the lack of standardization in the centrifugation method, cell counting, and data evaluation [5].

### 3.2. Standardization Efforts

To improve standardization and outline the most relevant parameters for the quality of each bone marrow-derived biological product, Lana and Purita, in 2020, evaluated the biological value of bone marrow aspirate clot as a feasible orthobiologic in musculoskeletal health and proposed the ‘ACH’ classification system, encompassing BMA (A), BMA concentrate (C), and hybrid (H), combining A and C. This classification aids in studying and using these techniques [35]. Everts et al., in 2020, compared the cellular and quality differences between a non-processed, direct application low-volume BMA preparation (10 mL), and a BMAC preparation, following centrifugal density separation of 60 mL of aspirated bone marrow. Laboratory analysis revealed that BMAC preparations highly significantly increased the cell concentrations of MSCs, platelets, TNCs, and CD34+ cells, with a significant decrease in erythrocytes. Furthermore, the cell viability between unprocessed BMA and processed BMAC was not statistically different [36].

In another study by Mautner et al. in 2020, the goal was to evaluate bone marrow aspiration techniques by comparing the number of MSCs, measured with CFU-f determinations, via low-volume bilateral PSIS aspiration with high-volume BMA extraction from unilateral PSIS draws, to address the most common locations of MSCs within the trabecular bone marrow. The study concluded that multi-site bone marrow aspirations of lower volumes result in higher concentrations of CFU-Fs [37]. In 2021, Lana et al. introduced a technique for using BMA clot mixed with hyaluronic acid, named BMA matrix. This innovative technique showed applicability and advantages over BMAC and PRP, detailing protocols and preparation steps. The BMA matrix is an easily reproducible and feasible technique, also serving to house transplanted cells and recruit circulating cells because of your scaffold [38].

Dwyer, in 2021, published an article demonstrating the efficacy of BMA in improving pain and quality of life in patients with glenohumeral osteoarthritis. The study compared BMA use with corticosteroids in 29 shoulders. This research further solidifies the regenerative potential of bone marrow-derived products when contrasted with conventional therapeutic approaches [47]. In 2022, Salamanna conducted a comparison between the bone marrow of young and old patients to evaluate the parameters of the mesenchymal cells in the BMA clot and ascertain if there were age-related disparities. The study found that the BMA clot preserves the regenerative properties of mesenchymal cells, regardless of the donor’s age. This suggests that bone marrow clots may be a viable source of mesenchymal cells for therapeutic applications in bone regeneration, especially in elderly patients [39].

Salamanna et al. also investigated the use of BMA clots in spinal fusion surgery, demonstrating its efficacy in enhancing spinal fusion rate and postoperative outcomes. Patients who underwent the adjunct technique of applying BMA clot experienced less pain and improved quality of life [28]. Additionally, they exhibited improvements in bone density and increased bone regeneration and stability alongside spinal fusion. This pilot study, conducted in 2020 with just 10 patients, showed a 100% efficacy of the technique [40]. In 2023, the same research group described the potential antimicrobial capabilities of BMA clots, showing that in addition to enhancing stability and bone regeneration, this technique could reduce the risks of postoperative infection. The study collected the evidence that demonstrates that the mesenchymal cells present in the BMA are capable of producing antimicrobial proteins and molecules such as cathelicidin LL-37 and hepcidin [41]. In the same year, Salamanna also published an article that provided important evidence on gender-based differences in the biology of MSCs derived from bone marrow clots [48]. Furthermore, the process variables that affect the yield of the MSCs were outlined by Muthu et al. in 2023 [49,50]. Later, clinical studies on the dose stratification of BMA in osteoarthritis were given by Jeyaraman et al. and Muthu et al. in 2024 as shown in Figure 2 [42,51].

Recently, several clinical trials have been conducted showing promising results with the use of BMA for treating femoral head osteonecrosis [52], osteoarthritis [53], osteochondritis dissecans [54], Achilles tendon rupture [54], hip disorders [55], and O’Donoghue’s Triad [56]. Furthermore, when searching on 9 May 2024, for clinical trials on ‘bone marrow and musculoskeletal diseases’ on clinicaltrials.gov (accessed on 9 May 2024), 72 studies were found, of which only three (NCT03410355, NCT03579407, and NCT01123850) were terminated and only one of these posted the results. Thus, further studies are warranted to delve deeper into the regenerative potential in these pathological conditions. Nonetheless, the preliminary results suggest the possibility of an alternative route preceding surgical intervention for musculoskeletal disorders. This avenue aims to alleviate pain, enhance quality of life, and circumvent surgical procedures through tissue regeneration.

## 4. Challenges

The evidence presented in this temporal overview indicates the considerable regenerative potential of bone marrow products across different forms and applications, right from the initial approaches and investigations. Significant progress has been made in exploring their characteristics and the mechanisms facilitating efficient tissue regeneration, extending beyond musculoskeletal tissue applications. This progress is also evident in a doubling of the number of articles about bone marrow-based therapies across all fields of medicine published during the past 10 years in PubMed. Scopus and Web of Science. Furthermore, the pace at which cell-based clinical trials for musculoskeletal indications are being registered has experienced exponential growth, surpassing the comparatively linear rise in the number of overall registered trials across all indications. This trend aptly mirrors the substantial demand for and enthusiasm surrounding regenerative therapies. It also underscores the considerable promise generated within the domain of cellular therapies, fueled by ongoing advancements in stem cell biology. Nevertheless, numerous aspects remain to be considered for a comprehensive understanding of these potent, readily available, cost-effective, and versatile tools in regenerative medicine. Recent systematic reviews on bone marrow cellular therapies in orthopedic clinical trials revealed specific deficiencies in reporting and characterizing cell populations. Both researchers and clinicians are increasingly recognizing the necessity for more rigorous characterization of cell sources, harvesting methods, processing techniques, and the composition of cell populations. As cell-based clinical trials continue to proliferate, there is an expectation for a greater emphasis on comparing alternative cell sources with clearly defined critical quality attributes. Addressing this gap presents an opportunity to enhance the granularity of available data, which would be invaluable for ongoing tracking and communication within the field.

### 4.1. Limitations

One of the key limitations of this paper is the lack of standardized methodologies in bone marrow cellular therapy research. While the paper acknowledges the need for uniform protocols in harvesting, processing, and applying bone marrow-derived products, it does not provide comprehensive solutions to address this issue. The variability in techniques across studies complicates the reproducibility of outcomes, which is crucial for validating the efficacy and safety of these therapies. Another limitation is the paper’s limited focus on comparative studies. It mainly concentrates on the advantages of bone marrow-derived products without thoroughly comparing them to other regenerative therapies, such as adipose-derived stem cells or platelet-rich plasma (PRP). Including such comparisons would provide a more comprehensive perspective on how bone marrow cellular therapies stand in relation to other available treatment options. Furthermore, the paper lacks an in-depth analysis of long-term outcome data. While it discusses recent clinical trials and their promising results, the absence of substantial long-term follow-up data means there is limited insight into the sustained efficacy and potential side effects of these therapies. Long-term outcomes are critical for fully understanding the therapeutic potential of bone marrow cellular products in managing musculoskeletal disorders. The paper also underexplores the specific molecular mechanisms behind the regenerative potential of mesenchymal stem cells (MSCs). Although it provides a broad overview of their biological properties, a more detailed investigation into the underlying molecular and cellular processes that drive tissue repair and regeneration, particularly in different musculoskeletal conditions, would enhance the scientific depth of the review. The paper provides limited discussion on the regulatory and ethical challenges associated with the clinical use of MSC-based therapies. Regulatory hurdles play a significant role in the translation of research into clinical practice, and addressing these aspects would have been valuable in offering a complete view of the current state and future directions of bone marrow cellular therapies.

### 4.2. Future Prospects

Despite the significant progress made in the field, several challenges remain. The need for rigorous characterization of cell sources, harvesting methods, processing techniques, and the composition of cell populations is critical. Recent systematic reviews have highlighted deficiencies in reporting and characterizing cell populations in clinical trials, underscoring the need for standardized protocols and methodologies. Future research should focus on comparing alternative cell sources with clearly defined critical quality attributes. This will enhance the granularity of the available data and improve the understanding of the regenerative potential of different cell populations. Additionally, exploring the integration of bone marrow-derived products with other regenerative medicine techniques, such as gene therapy and tissue engineering, could open new avenues for treating musculoskeletal disorders. The promising avenues for future research include the development of non-surgical interventions that leverage the regenerative properties of bone marrow-derived products. These approaches aim to alleviate pain, enhance quality of life, and reduce the need for surgical procedures through tissue regeneration and repair. It is crucial to establish rigorous standardization in cell source identification, harvesting methods, and processing techniques to ensure consistency and improve data quality in clinical trials. Developing uniform protocols for characterizing and reporting cell populations is essential. Defining critical quality attributes for alternative cell sources will allow for better comparison across studies and treatments. Investigating the integration of bone marrow-derived products with advanced regenerative approaches, such as gene therapy, tissue engineering, and potentially CRISPR-based methods, could enhance the therapeutic potential of these treatments. This integration may open new avenues for more effective management of musculoskeletal disorders. Future research should prioritize non-surgical interventions using bone marrow-derived products to harness their regenerative properties, thereby improving patient outcomes through pain reduction, enhanced quality of life, and reduced need for surgical procedures. Clinical trials must also focus on long-term outcomes, safety profiles, and scalability to facilitate the broader adoption of these therapies in clinical practice. Exploring new pathways and molecular mechanisms that underlie the regenerative potential of mesenchymal stem cells (MSCs) will be essential for refining and optimizing their therapeutic applications.

## 5. Conclusions

In tracing the historical trajectory and contemporary advances in bone marrow cellular therapy for musculoskeletal disorders, this review highlights the profound impact of foundational research and modern innovations in the field. From the pioneering work of Friedenstein in the late 1960s to the sophisticated clinical applications seen today, bone marrow-derived mesenchymal stem cells (MSCs) have consistently demonstrated their potential in promoting tissue repair and regeneration. The historical context provided a critical understanding of the biological properties and initial applications of MSCs, setting the stage for significant breakthroughs. Contemporary research has focused on optimizing isolation and application techniques, ensuring higher efficacy and safety in clinical settings. Innovations such as the ACH classification system and advanced aspiration methods have standardized practices, leading to more reliable and reproducible outcomes. Clinical studies have validated the therapeutic benefits of bone marrow-derived products across a range of musculoskeletal conditions, from osteonecrosis to osteoarthritis. These advancements underscore the versatility and efficacy of MSCs in both surgical and non-surgical treatments, highlighting their role in reducing pain, enhancing tissue repair, and improving patient quality of life. Looking forward, the field must address challenges related to the rigorous characterization of cell populations and the standardization of clinical trial methodologies. Future research should continue to explore the integration of MSCs with other regenerative techniques, expanding their therapeutic potential and applications. The evolution of bone marrow cellular therapy marks a significant milestone in regenerative medicine. The continued exploration and refinement of these therapies promise to further revolutionize the treatment of musculoskeletal disorders, offering hope for improved patient outcomes and advancing the frontier of medical science.

## Figures and Tables

**Table 1:** Pioneering work in BMAC.

Year	Researcher(s)	Key Contributions
2014	Centeno [7]	Compared BMAC alone vs. BMAC with fat grafting for knee osteoarthritis and concluded that addition of fat grafting has not exhibited an additional regenerative effect than BMAC alone.
2017	Salamanna [33]	Conducted a systematic review on the use of BMA clot as a scaffold for tissue regeneration where he described the usage of the BMA clot in eight pre-clinical and three clinical studies and concluded that the BMA clot as a plausible scaffold for tissue regeneration.
2020	Lana and Fonseca [34]	Evaluated the biological value of bone marrow aspirate clot as a feasible orthobiologic in musculoskeletal health.
2020	Purita and Lana [35]	Proposed an ACH classification system for bone marrow-derived products, which emphasizes the quality control of bone marrow-derived products in clinical usage.
2020	Everts et al. [36]	Centrifugal density separation facilitates higher BMAC cellular yields than low-volume BMA where they described the factors responsible for higher BMAC cellular yields.
2020	Mautner et al. [37]	Multi-site low-volume BMA aspirations increase CFU-fs and other cells when compared to single-site high-volume aspirations.
2021	Lana et al. [38]	Introduced the BMA matrix technique mixed with hyaluronic acid where BMA matrix represents a suitable alternative, indicated for the enhancement of tissue repair mechanisms by modulating inflammation and acting as a natural biological scaffold as well as a reservoir of cytokines and growth factors that support cell activity.
2022	Salamanna [39]	Studied the age-related efficacy of BMA clot in bone regeneration and concluded that the donor age does not affect functional and phenotypical characteristics of clotted BMA.
2023	Salamanna [40]	Safety and efficacy of autologous bone marrow clot as a multifunctional bio-scaffold for instrumental posteriolateral lumbar fusion where the results indicate a successful posterolateral lumbar fusion rate of 100% at the 12-month follow-up, along with an increase in bone density from 6 to 12 months of follow-up.
2023	Contartese [41]	Ability of BMA clot to provide a local combined delivery system not only of stem cells, signalling biomolecules, and anti-inflammatory factors but also of molecules and proteins endowed with antimicrobial properties.
2024	Jeyaraman and Muthu [42]	Dose stratification of BMAC [minimal clinically important differences (MCID)—2 million BMAC cells per kilogram body weight] in the management of knee osteoarthritis.
