# Correction: Lana et al. Evolution and Innovations in Bone Marrow Cellular Therapy for Musculoskeletal Disorders: Tracing the Historical Trajectory and Contemporary Advances. *Bioengineering* 2024, *11*, 979

**DOI:** 10.3390/bioengineering12020161

**Published:** 2025-02-07

**Authors:** José Fábio Lana, Gabriela Caponero de Brito, André Kruel, Benjamim Brito, Gabriel Silva Santos, Carolina Caliari, Francesca Salamanna, Maria Sartori, Giovanni Barbanti Brodano, Fábio Ramos Costa, Madhan Jeyaraman, Ignácio Dallo, Pedro Bernaldez, Joseph Purita, Marco Antonio Percope de Andrade, Peter Albert Everts

**Affiliations:** 1 Department of Orthopaedics, Brazilian Institute of Regenerative Medicine (BIRM), Indaiatuba 13334-170, SP, Brazil; josefabiolana@gmail.com (J.F.L.); gabriela_caponero@hotmail.com (G.C.d.B.); kruel.andre@gmail.com (A.K.); contato@clinica4move.com.br (B.B.); 2 Regenerative Medicine, Orthoregen International Course, Indaiatuba 13334-170, SP, Brazil; doctorignaciodallo@gmail.com (I.D.); jpurita@aol.com (J.P.); everts@me.com (P.A.E.); 3 Medical School, Max Planck University Center (UniMAX), Indaiatuba 13343-060, SP, Brazil; 4 Clinical Research, Anna Vitória Lana Institute (IAVL), Indaiatuba 13334-170, SP, Brazil; 5 Medical School, Jaguariúna University Center (UniFAJ), Jaguariúna 13820-000, SP, Brazil; 6 Cell Therapy, In Situ Terapia Celular, Ribeirão Preto 14056-680, SP, Brazil; caliari.carolina@gmail.com; 7 Surgical Sciences and Technologies, IRCCS Instituto Ortopedizo Rizzoli, 40136 Bologna, Italy; francesca.salamanna@ior.it (F.S.); maria.sartori@ior.it (M.S.); 8 Spine Surgery Unit, IRCCS Instituto Ortopedizo Rizzoli, 40136 Bologna, Italy; giovanni@barbantibrodano.com; 9 Department of Orthopaedics, FC Sports Traumatology, Salvador 40296-210, BA, Brazil; fabiocosta123@uol.com.br; 10 Department of Orthopaedics, ACS Medical College and Hospital, Dr. MGR Educational and Research Institute, Chennai 600077, Tamil Nadu, India; madhanjeyaraman@gmail.com; 11 Orthopaedic Research Group, Coimbatore 641045, Tamil Nadu, India; 12 Clinical Research Scientist, Virginia Tech India, Chennai 600095, Tamil Nadu, India; 13 Orthopedics, SportMe Medical Center, 41013 Seville, Spain; pedrobernaldez@gmail.com; 14 Department of the Locomotor Apparatus, Federal University of Minas Gerais, Belo Horizonte 31270-901, MG, Brazil; mapa.bhz@terra.com.br; 15 Gulf Coast Biologics, Fort Myers, FL 33916, USA

## Error in Figure 2

In the original publication [1], there were errors in Figure 2. In group 2020 and 2021, there is a missing author’s name “Fonseca”. The corrections are to add the name “Fonseca” in groups 2020 and 2021. They should read as follows:

**Figure 2:**
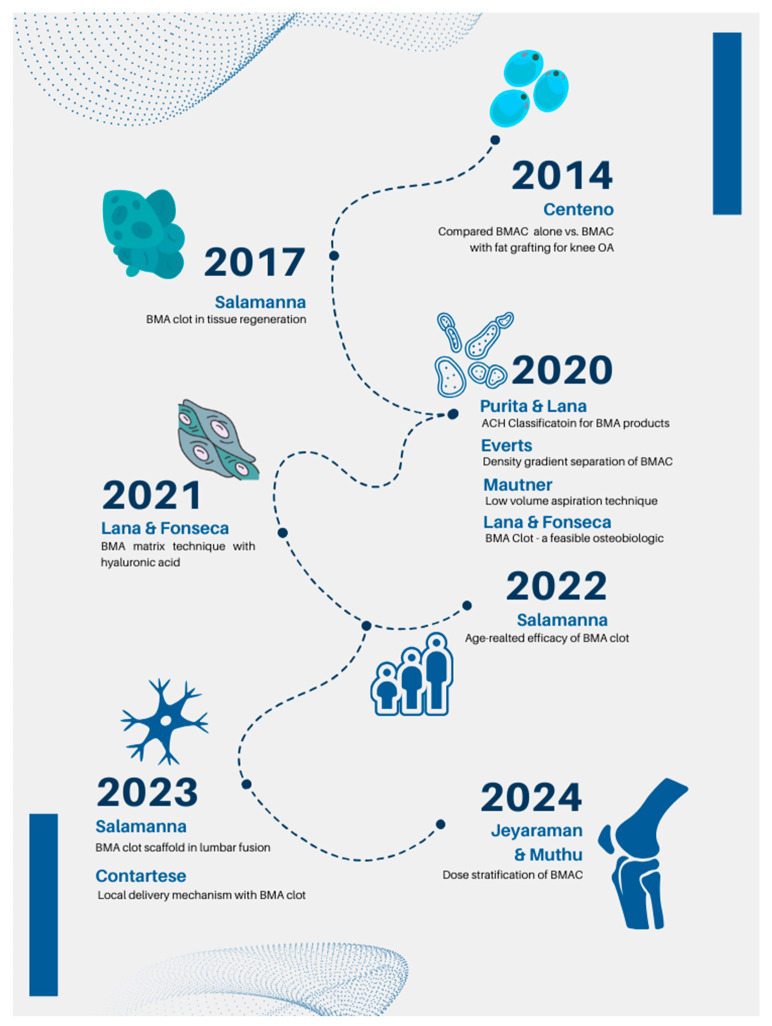
Recent clinical advances in the bone marrow cellular therapies.

## Missing Citation

In the original publication [1], reference [34] “Lana, J.F.S.D.; Fonseca, L.F.; Mosaner, T.; Tieppo, C.E.; Azzini, G.; Ribeiro, L.L.; Setti, T.; Purita, J. Bone Marrow Aspirate Clot: A Feasible Orthobiologic. *J. Clin. Orthop. Trauma.* **2020**, *11*, S789–S794.” was not cited in Table 1. The citation has now been inserted in Table 1 and the corresponding description has been added. They should read as follows:

With this correction, the order of some references has been adjusted accordingly. The authors state that the scientific conclusions are unaffected. This correction was approved by the Academic Editor. The original publication has also been updated.

## Figures and Tables

**Table 1:** Pioneering work in BMAC.

Year	Researcher(s)	Key Contributions
2014	Centeno [7]	Compared BMAC alone vs. BMAC with fat grafting for knee osteoarthritis and concluded that addition of fat grafting has not exhibited an additional regenerative effect than BMAC alone.
2017	Salamanna [33]	Conducted a systematic review on the use of BMA clot as a scaffold for tissue regeneration where he described the usage of the BMA clot in eight pre-clinical and three clinical studies and concluded that the BMA clot as a plausible scaffold for tissue regeneration.
2020	Lana and Fonseca [34]	Evaluated the biological value of bone marrow aspirate clot as a feasible orthobiologic in musculoskeletal health.
2020	Purita and Lana [35]	Proposed an ACH classification system for bone marrow-derived products, which emphasizes the quality control of bone marrow-derived products in clinical usage.
2020	Everts et al. [36]	Centrifugal density separation facilitates higher BMAC cellular yields than low-volume BMA where they described the factors responsible for higher BMAC cellular yields.
2020	Mautner et al. [37]	Multi-site low-volume BMA aspirations increase CFU-fs and other cells when compared to single-site high-volume aspirations.
2021	Lana et al. [38]	Introduced the BMA matrix technique mixed with hyaluronic acid where BMA matrix represents a suitable alternative, indicated for the enhancement of tissue repair mechanisms by modulating inflammation and acting as a natural biological scaffold as well as a reservoir of cytokines and growth factors that support cell activity.
2022	Salamanna [39]	Studied the age-related efficacy of BMA clot in bone regeneration and concluded that the donor age does not affect functional and phenotypical characteristics of clotted BMA.
2023	Salamanna [40]	Safety and efficacy of autologous bone marrow clot as a multifunctional bio-scaffold for instrumental posteriolateral lumbar fusion where the results indicate a successful posterolateral lumbar fusion rate of 100% at the 12-month follow-up, along with an increase in bone density from 6 to 12 months of follow-up.
2023	Contartese [41]	Ability of BMA clot to provide a local combined delivery system not only of stem cells, signalling biomolecules, and anti-inflammatory factors but also of molecules and proteins endowed with antimicrobial properties.
2024	Jeyaraman and Muthu [42]	Dose stratification of BMAC [minimal clinically important differences (MCID)—2 million BMAC cells per kilogram body weight] in the management of knee osteoarthritis.
